# Two inflammation-related genes model could predict risk in prognosis of patients with lung adenocarcinoma

**DOI:** 10.1007/s12094-025-03861-w

**Published:** 2025-03-07

**Authors:** Wei Yang, Junqi Long, Gege Li, Jiashuai Xu, Yining Chen, Shijie Zhou, Zhidong Liu, Shuangtao Zhao

**Affiliations:** 1 Department of Thoracic Surgery, Beijing Tuberculosis and Thoracic Tumor Research Institute/Beijing Chest Hospital, Capital Medical University, Beijing, 101149 China; 2 School of Software Engineering, Faculty of Information Technology, Beijing University of Technology, Beijing, 100124 China

**Keywords:** LUAD, Prognosis, Inflammation-related genes, Cox regression

## Abstract

**Background:**

In lung adenocarcinoma (LUAD), there remains a dearth of efficacious diagnostic studies including some inflammation-related genes to identify the LUAD subgroups with different clinical outcomes.

**Methods:**

First, two molecular subgroups were identified with mRNA expression profiling from The Cancer Genome Atlas (TCGA) by K-means algorithm. Gene set enrichment analysis (GSEA), immune infiltration, and Gene set variation analysis (GSVA) were applied to explore the biological functions between these two subtypes. Then, univariate and multivariate Cox regression analyses were selected to evaluate the independence of these subtypes in LUAD. Next, lasso regression was applied to identify the high-precision mRNAs to predict the subtype with favorable prognosis. Finally, a two-mRNA model was constructed using the method of multivariate Cox regression, and the effectiveness of the model was validated in a training set (*n* = 310) and three independent validation sets (*n* = 1.

**Results:**

Comprehensive genomic analysis was conducted of 310 LUAD samples and identified two subtypes associated with molecular classification and clinical prognosis: immune-enriched and non-immune-enriched subgroup. Then, a new model was developed based on two mRNAs (*MS4A1* and *MS4A2*) in TCGA dataset and divided these LUAD patients into high-risk and low-risk subgroup with significantly different prognosis (HR = 1.644 (95% CI 1.153–2.342); *p* < 0.01), which was independence of the other clinical factors (*p* < 0.05). In addition, this new model had similar predictive effects in another three independent validation sets (HR > 1.445, *p* < 0.01).

**Conclusions:**

We constructed a robust model for predicting the risk of LUAD patients and evaluated the clinical outcomes independently with strong predictive power. This model stands as a reliable guide for implementing personalized treatment strategy.

**Supplementary Information:**

The online version contains supplementary material available at 10.1007/s12094-025-03861-w.

## Introduction

In recent years, lung cancer has become one of the most common cancers globally with a high mortality rate [1]. Non-small cell lung cancer (NSCLC) is the major subtype, accounting for approximately 85% of all cases [2–4]. Adenocarcinoma is the major histologic subtype of NSCLC, accounting for about 60% of NSCLC [5, 6]. The LUAD subtype accounts for the highest number of deaths among all subtypes in the clinical practice [1, 7, 8]. In the clinical, LUAD lacks distinctive clinical features [9] and possesses a low survival rate and a high recurrence rate [1, 10–13]. Therefore, there is an urgent need to develop new molecular prognostic biomarkers to predict the prognosis risk of LUAD patients for targeted therapies and personalized precision treatments [14].

Currently, an increasing number of studies have discovered that effectively identifying potential prognostic biomarkers could contribute to a deeper understanding of the biological mechanisms of cancer and stratify patients effectively based on prognosis risk. Jiang *et al*. constructed a prognostic prediction model based on ferroptosis genes in LUAD [15]. Yang et al. developed an overall survival (OS) prediction model for LUAD patients based on long non-coding RNA [16]. Wu et al. developed an OS prediction model for LUAD patients based on the tumor microenvironment [17]. Zhang et al. constructed a risk prognosis model for LUAD based on T cells [18]. Wang and Chen respectively constructed prognostic models for LUAD patients based on cuproptosis-related genes [19, 20]. Huang constructed a prognostic model for LUAD patients based on disulfidptosis-related genes [21]. Although some predictive capabilities were observed in the above studies, they are not sufficient for a precise understanding of the biological functional mechanisms of LUAD samples. Additionally, some prognostic feature extractions are complex, and there is a certain degree of gene redundancy.

Inflammation is associated with lung cancer and plays an important role in cancer progression [22–24]. Some studies suggest that assessing inflammatory markers in the blood can be used to evaluate the prognosis of patients. For example, Moik et al. demonstrated that clinical indicators, such as platelet–lymphocyte ratio (PLR), neutrophil–lymphocyte ratio (NLR), and lymphocyte–monocyte ratio (LMR), have strong predictive capabilities for overall survival (OS) [25]. And research indicates that inflammation can promote cancer cell proliferation and metastasis [26–28]. For instance, pro-inflammatory cytokines, such as TNFα, IL-6, IL-1a, and IL-1β, can facilitate anticancer responses [29]. Therefore, to deeply understand the biological function mechanism of LUAD, it is necessary to study inflammation-related genes and construct a stable risk diagnostic model based on mRNA. In this study, we developed a two-mRNA model including two inflammation-related genes with Cox regression algorithm to assess prognosis independently in the TCGA dataset. And then we validated the generalization ability of this new model in the three testing sets.

## Methods

### Identification of two molecular subgroups

First, a total of 978 candidate genes were screened from 1,027 inflammation-related genes [30]. Then, the 301 inflammation-related genes with cancer were screened using Deseq2 [31] between lung adenocarcinoma and normal samples. Finally, lung adenocarcinoma patients were classified into two subgroups using k-means, and the classification results were validated through the survival analysis and the principal component analysis (PCA).

### Identification of enriched signaling pathways

Gene Set Enrichment Analysis (GSEA) was performed by 71 DEGs between the two subtypes and gene sets from the Molecular Signature Database (MSigDB) [32] to identify signaling pathways that were significantly enriched (FDR *q*-value ≤ 0.05). In addition, the biological functions of these DEGs were verified by Gene Set Variation Analysis (GSVA) analysis [33].

### Immune infiltration analysis

Two deconvolution methods were used to assess the immune infiltration in the LUAD samples. The first method used MCP-counter [34] to generate absolute abundance scores for the eight major immune cell types, fibroblasts, and endothelial cells. The second method was used to assess the relative cell proportions of immune cell types using the CIBERSORT algorithm [35].

### Statistical analysis

We used the Fisher’s test to analyze the significance of clinical factors between the two subtypes. In addition, survival independence of the two subtypes and risk models was tested using survival analysis, Cox regression analysis, and multivariate Cox regression analysis [36]. Finally, these results were visualized using the R packages *ggplot2* [37] and forestplot [38].

### Screening the optimal feature genes

To avoid the problem of gene covariance, candidate genes with the best prognostic performance were identified by the immune gene set and Lasso survival regression analysis [39] among 71 Differentially Expressed Genes (DEGs) between two different subgroups. In addition, we performed immunohistochemistry (IHC) validation of tumor marker gene expression from The Human Protein Atlas (THPA, https://www.proteinatlas.org/) LUAD samples.

### Training data and validation data

**Fig. 1:**
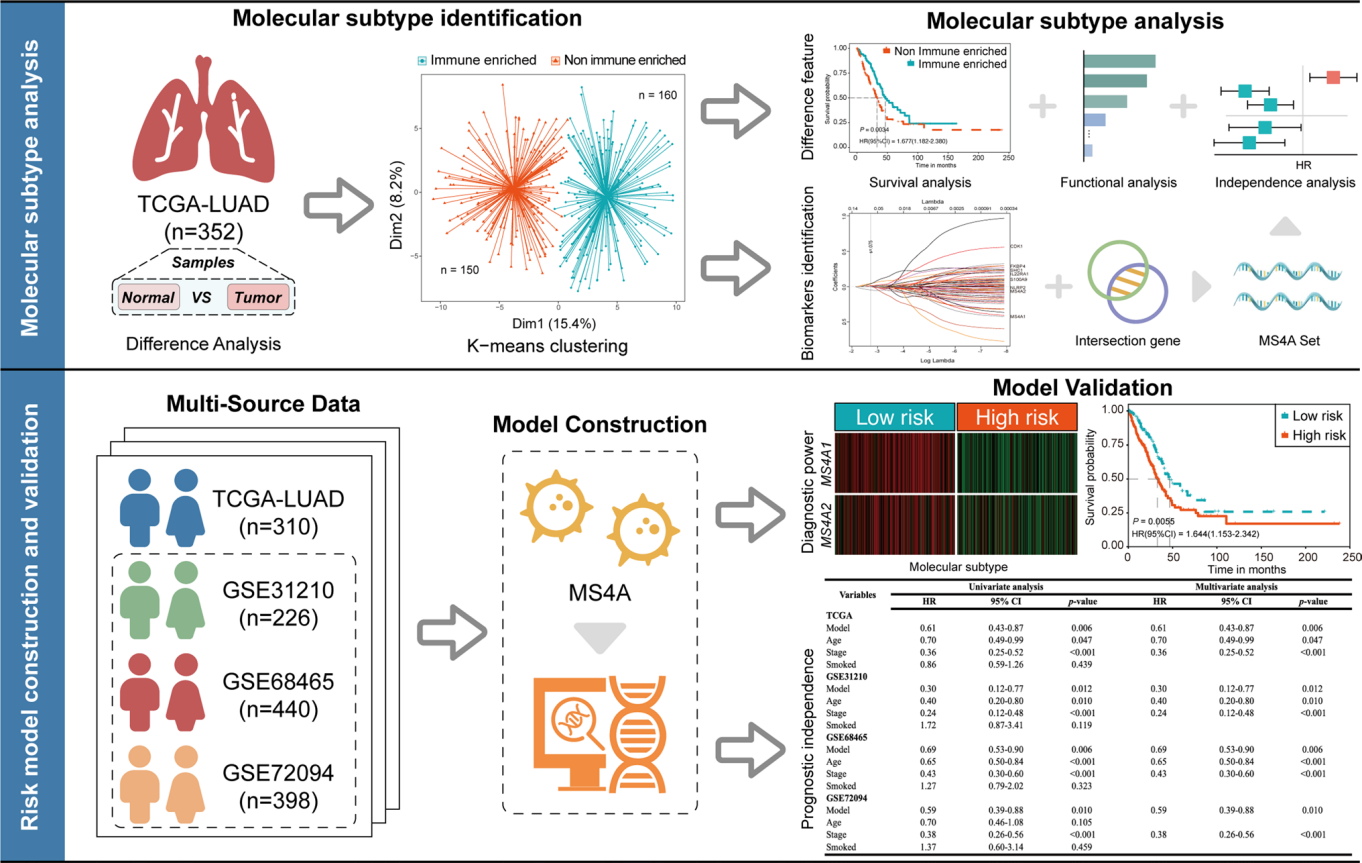
Flowchart of this study. The overall research workflow primarily consists of two major parts: the first part involved the identification and analysis of molecular subtypes, while the second part focused on the construction of a prognostic risk model and multidimensional validation

The original files were downloaded from the TCGA (https://portal.gdc.cancer.gov) and GEO (https://www.ncbi.nlm.nih.gov/geo) databases. Patients from TCGA were considered as the training set, while patients from GSE31210, GSE68465, and GSE72094 were considered as three validation sets (Table s1). In the TCGA data, we selected 42 normal samples and 310 lung adenocarcinoma samples. In the GEO data, a total of 1,064 lung adenocarcinoma samples were selected for the subsequent research and validation. The entire study flow is displayed in Fig. 1.

## Results

### Identification of two molecular subgroups in LUAD

**Fig. 2:**
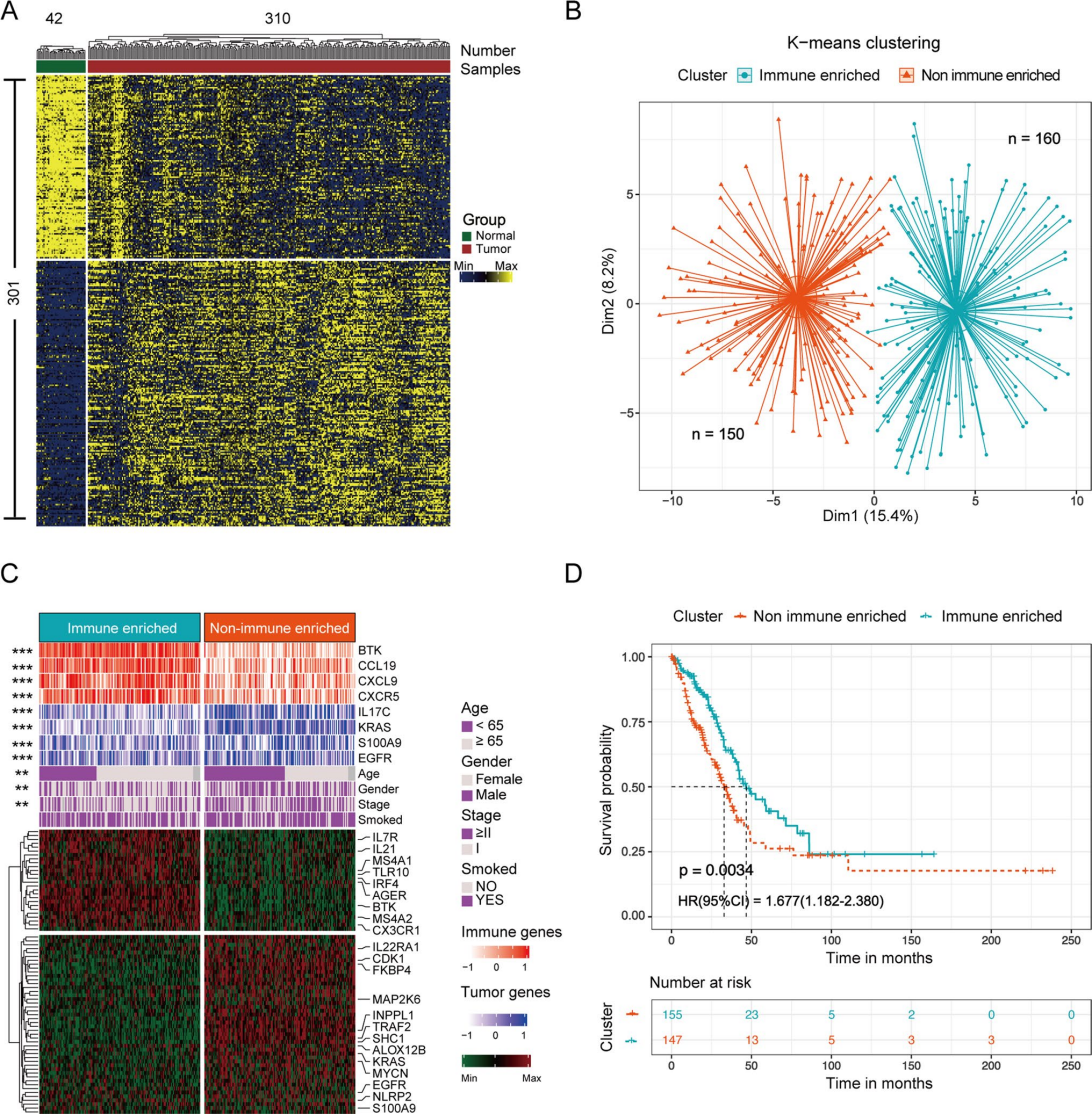
Identification of two molecular subgroups in LUAD. **A** Analysis of genomic differences between normal and tumor patients based on 978 inflammatory genes. **B** Two clusters were identified within 310 LUAD patients by the k-means algorithm. **C** Unsupervised hierarchical clustering was performed in 310 samples (*k* = 2) using the 71 DEGs within RNA expression values in the TCGA cohort, and the names of cancer or immune-related genes were labeled on the right. Clinical covariates were shown in the heatmap above. Fisher’s exact test: * < 0.05, ** < 0.01, *** < 0.001. **D** Differences in patient overall survival between the two subtypes (log-rank *p*)

To understand the mechanistic role of inflammatory genes in LUAD patients, we filtered 301 inflammatory DEGs between normal (*n* = 42) and tumor (*n* = 310) samples in TCGA for subsequent analysis (Fig. 2A). We identified the two optimal classifications with significant differences by unsupervised k-means clustering of 310 tumor samples and defined them as immune-enriched and non-immune-enriched subtypes (Figs. 2B & S1A). To explore the differences in gene expression profiles between different subgroups, we conducted a differential analysis between the two subgroups. A total of 71 inflammatory-related DEGs were identified with a threshold of |log2FC|> 1 and adj.*p* < 0.05, of which 26 genes were overexpressed in immune-enriched group and 45 genes in non-immune-enriched group (Figs. 2C & S1B). And, we also analyzed differences in clinical factors (such as age, gender, clinical status, and smoked), immune marker genes (including *BTK*, *CCL19*, *CXCL9*, *CXCR5*), and cancer marker genes (such as *IL17C*, *KRAS*, *S100A9*, *EGFR*) between the two subtypes, the results showed that all clinical factors and marker genes except smoked factor in the two subtypes had significant differences (*p* < 0.01). In addition, survival analysis revealed a significant difference in clinical survival between the immune-enriched and non-immune-enriched groups, with immune-enriched group patients exhibiting more favorable survival (*p* = 0.003, HR (95% CI) = 1.677(1.182–2.380), Fig. 2D). The results of all the analyses proved that our subtypes had significant effect on the prognosis of LUAD patients.

### Different features between two molecular subgroups

**Fig. 3:**
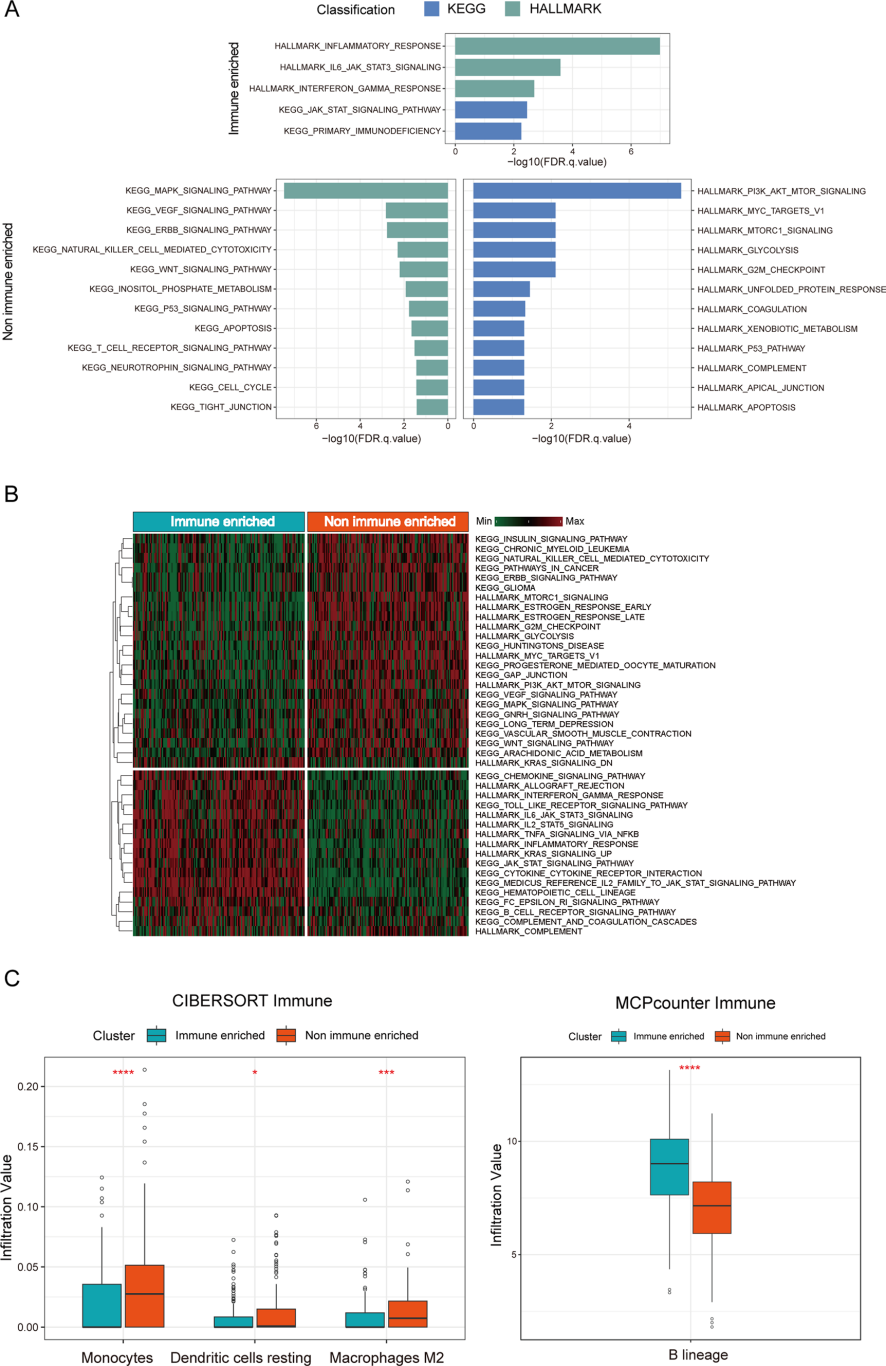
Different features between two molecular subgroups. **A** Pathway enrichment analysis identified biological pathways enriched in immune and non-immune-enriched group. The curated gene sets were downloaded from the Molecular Signatures Database (MSigDB). Both the cancer Hallmark and KEGG gene sets were shown. FDR *q*-value, the *p*-value adjusted for the false discovery rate (FDR). A *q*-value threshold of 0.05 (5% FDR) was applied. **B** The significant biological pathways were displayed between immune and non-immune-enriched subgroup determined by Gene Set Variation Analysis. **C** Immune infiltration expression values between immune and non-immune-enriched subtype were inferred by CIBERSORT and MCP-counter algorithm

To understand the distinct biological function between these two subgroups, we performed the pathway enrichment analysis to identify molecular function within the genomic data (Fig. 3A). The results revealed that five significantly upregulated signaling pathways in the immune-enriched group, primarily composed of the immune-correlated pathways, such as inflammatory response, IL-6-JAK-STAT3 signaling, interferon gamma response, and so on. Similarly, a total of 24 upregulated pathways were identified in the non-immune-enriched group, primarily associated with cell cycle and cancer response signal functions, including G2M checkpoint, cell cycle, MAPK signaling, PI3K-AKT-MTOR signaling, etc. To further validate the biological characterization of these two subtypes, we applied the GSVA algorithm into calculate the biological function of each subtype, which was functionally consistent with the subtypes described above (Fig. 3B). Additionally, we utilized two deconvolution methods to analyze the immune cell infiltration scores between these two subtypes. Monocytes, dendritic cells resting, and macrophages M2 showed the significant differences in the CIBERSORT immune cell type assessment and higher expression in the non-immune-enriched group. Similarly, B lineage expression was higher in the immune-enriched group in the MCP-counter method assessment. It could be observed that the functional analysis was consistent across subtypes: monocytes, dendritic cells resting, and macrophages M2 inhibited immune progression, and B lineage promoted immune progression (Fig. 3C).

### Independent prognostic evaluation of different molecular subtypes

**Fig. 4:**
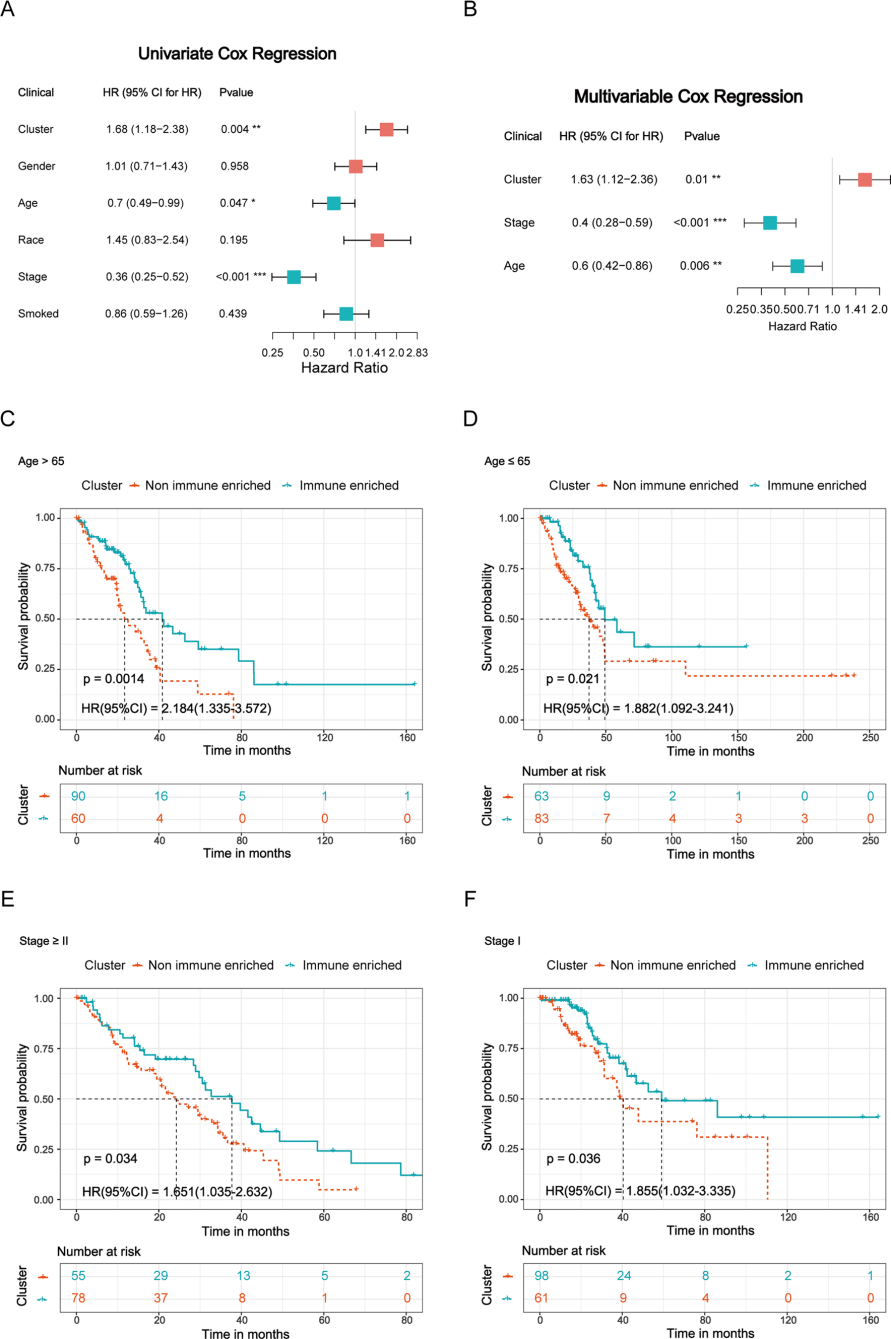
Independent prognostic evaluation of different molecular subtypes **A** Univariate cox regression analyses between 5 clinical factors and 2 subtypes. **B** Multivariate cox regression analyses between 5 clinical factors and 2 subtypes. **C**–**F** Kaplan–Meier survival analysis of all LUAD patients in TCGA according to subtypes stratified by significant clinicopathologic risk factors

To assess the independence of this subtype in predicting prognosis, we conducted univariate and multivariate Cox regression analysis within some clinical factors including patients’ age, race, gender, clinical stage, and smoked status. We discovered that the cluster, the age, and the clinical stage were significant in both univariate and multivariate Cox regression analyses (*p* < 0.05, Fig. 4A, B). Next, the stratified analyses were performed in the two clinical factors (clinical stage and age) based on the results above to further validate the independence of the subtype. As a result, this cluster significantly stratified patients into two subgroups with age > 65, age ≤ 65 or stage ≥ II, and stage I (*p* = 0.001, 0.021, 0.034 and 0.036, respectively; Fig. 4C–F). Therefore, all the results indicated that this cluster was an independent prognosis biomarker among all those clinical factors.

### Optimal feature genes selection for two molecular subtypes

**Fig. 5:**
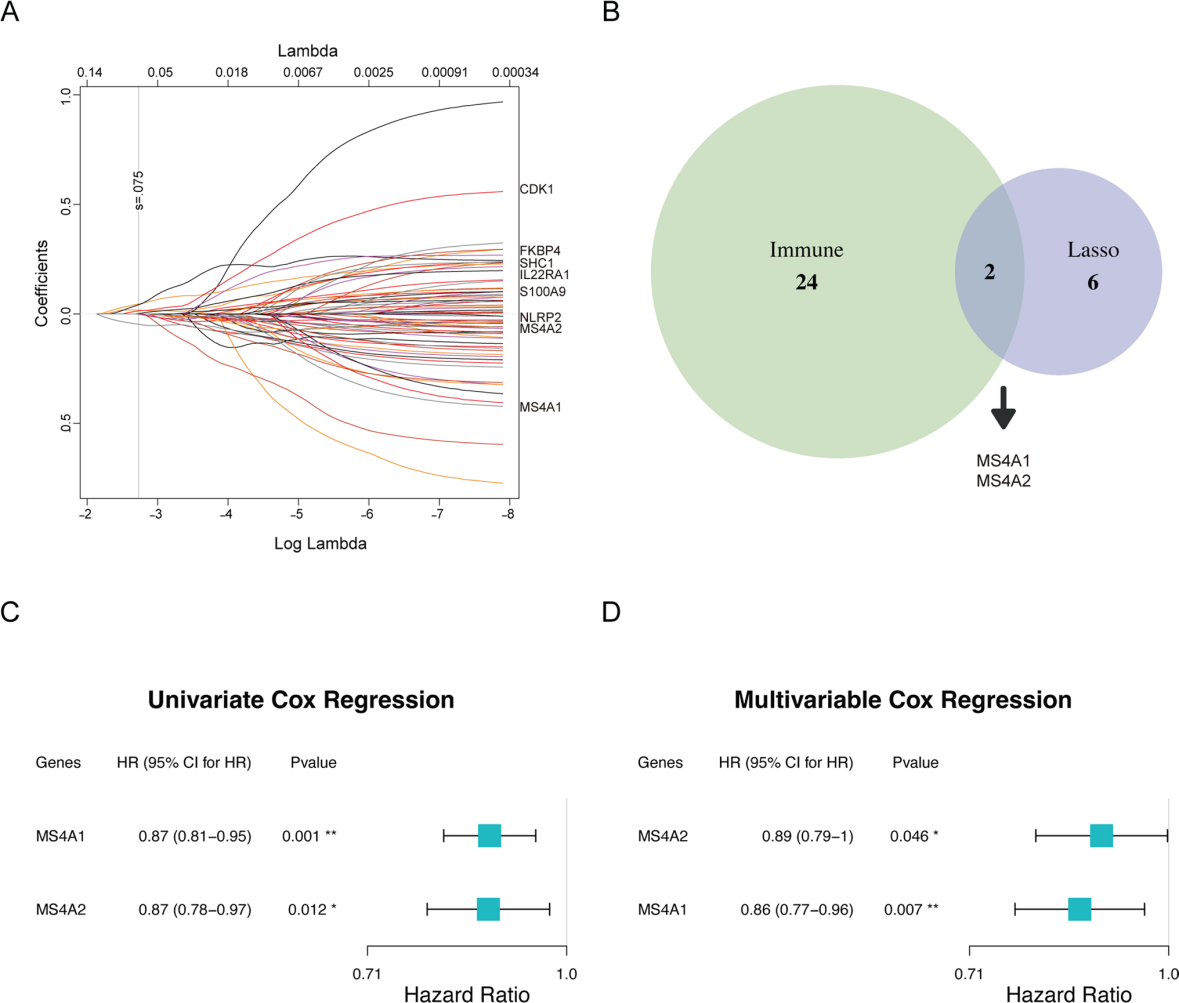
Optimal feature genes were selected to construct a 2-gene model. **A** The survival-correlated different expression genes (DEGs) were screened with LASSO coefficient profiles. **B** Overlaps between genes selected by Lasso survival analysis and enrichment in two subtypes. **C**, **D** Independence of optimal feature genes verified by univariate and multivariate Cox regression analyses

To identify the optimal feature genes without collinearity for the new subtypes, we initially selected LASSO survival analysis to screen the 71 inflammation-related genes. The results showed that these 8 genes (*FKBP4*, *IL22RA1*, *MS4A1*, *SHC1*, *CDK1*, *MS4A2*, *NLRP2*, and *S100A9*) were identified to be the most significant impact on survival when lambda reached the minimum value of 0.0745 (Figs. 5A & S1C). Then, two immune inflammation-related genes (*MS4A1*, *MS4A2*) were identified as the critical factors for classification in the immune-enriched group with favorable prognosis (Fig. 5B). Finally, the univariate and multivariate Cox regression analyses were completed between these two genes in the TCGA cohort to further validate the significantly predictive efficacy in clinical survival (*MS4A1* (HR = 0.87, 95% CI 0.81–0.95, *p* = 0.001), and *MS4A2* (HR = 0.87, 95% CI 0.78–0.97, *p* = 0.012); Fig. 5C), and similarly in the multivariate analysis (*MS4A1* (HR = 0.86, 95% CI 0.77–0.96, *p* = 0.007), *MS4A2* (HR = 0.89, 95% CI 0.79–1, *p* = 0.046; Fig. 5D). In addition, we found that the gene expression levels of *MS4A1* and *MS4A2* [40] were significantly higher in tumor tissues compared to normal tissues (Fig. S1D) and LUAD patients with higher expression levels exhibited better prognostic [41] based on existing IHC studies and experimental data, which is consistent with the above studies. Generally, these results pointed out that these two genes could discriminate the lung cancer patients within two subgroups with different prognosis.

### Construction and validation of the mRNA-based risk model

**Fig. 6:**
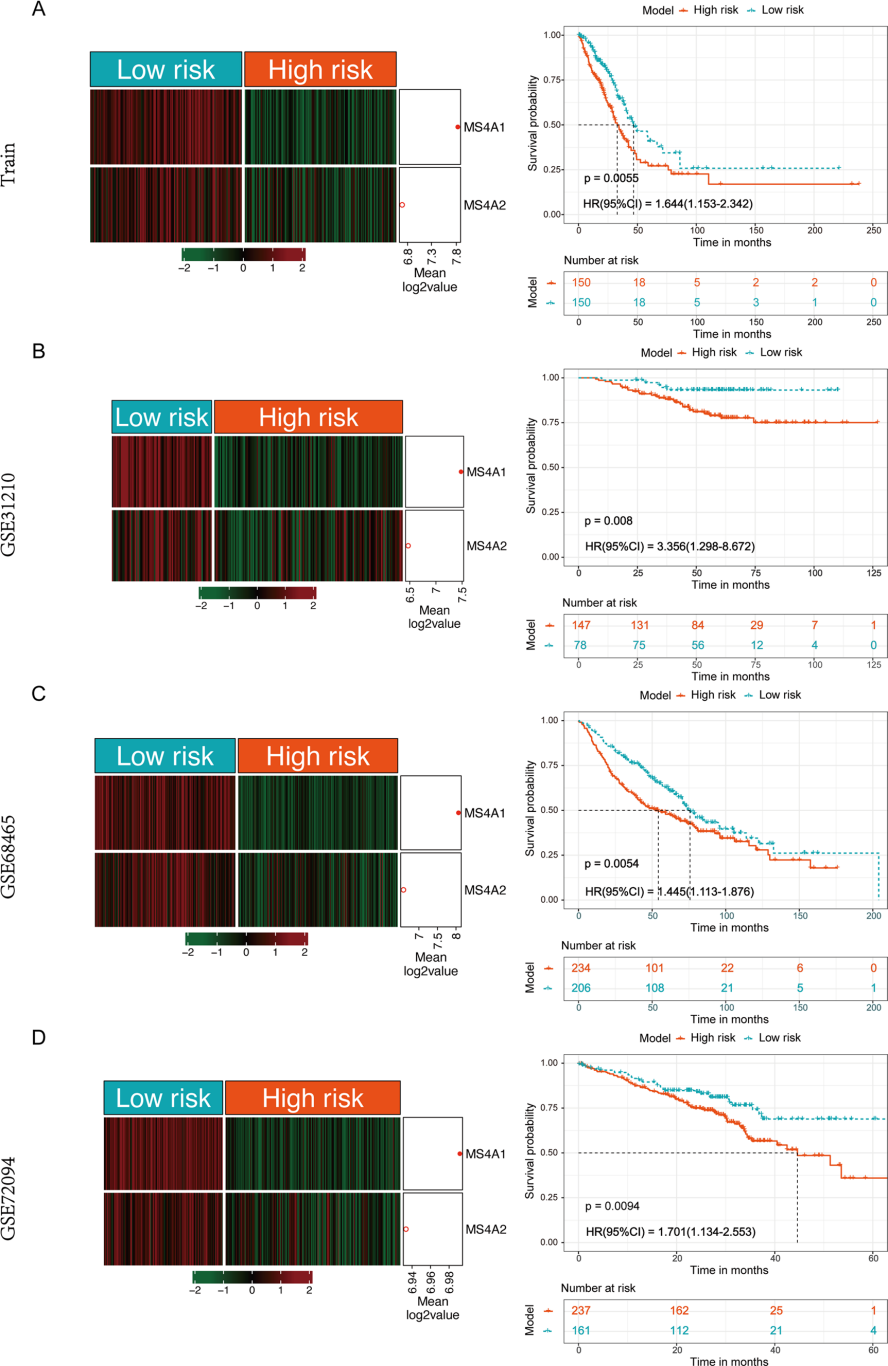
Risk stratification by the 2-gene model, and overall survival analysis by Kaplan–Meier method between high and low-risk group. **A** Training cohort. **B**–**D** Another 3 independent testing cohorts. *CI* confidence interval; *HR* hazard ratio

To effectively predict the survival risk of all patients, we constructed a 2-gene model using the identified key survival genes: risk score = – 0.12 * *MS4A2* – 0.15 * *MS4A1* and stratified all samples into high and low-risk group using the median risk score (− 2.005) as a cutoff value in the training set (*n* = 310). The results showed that the mRNA expression of these two genes in lung cancer patients within high-risk group was significantly lower than those within low-risk group (*p* < 0.001, Fig. 6A). The prognosis of the lung cancer patients from high-risk group was significantly poorer than that in low-risk group (*p* = 0.006, HR = 1.644 (95% CI 1.153–2.342), Fig. 6A). Then, to validate its stability, this new model was applied into another three separate external validation sets of lung adenocarcinoma (*n* = 1064): GSE31210 (*n* = 226), GSE68465 (*n* = 440), GSE72094 (*n* = 398), respectively. It was observed that the three validation sets could be significantly categorized into high and low-risk groups (*p* < 0.01; Fig. 6B–D), and the risk model could successfully divide all samples into two subgroups with significantly prognostic heterogeneity (*p* = 0.008, HR = 3.356 (95% CI 1.298–8.672) in GSE31320; *p* value = 0.005, HR = 1.445 (95% CI 1.113–1.876) in GSE68465; *p* = 0.009, HR = 1.701 (95% CI 1.134–2.553) in GSE72094; Fig. 6B–D), which was highly consistent with the results of the survival analysis in the training set. Additionally, we compared recently published prognostic prediction models for LUAD patients [42–47], it is noteworthy that the two MS4A family gene risk scores were the most streamlined prognostic diagnostic model for LUAD patients (*p* < 0.01, Fig. S1E), which has relatively novel study value. All the results suggested that this new model could identify the clinical outcome of lung cancer patients powerfully with high- and low-risk values.

### Prognostic prediction by the 2-gene model was independent of clinicopathological factors

To confirm the independence of this new 2-gene model in predicting prognosis, a total of 1374 patients were enrolled into a univariate and multivariate Cox regression analyses from a training set and another three testing sets. The results demonstrated this 2-gene model significantly correlated with prognosis when adjusted for the other 3 clinical factors in each dataset (HR < 0.7, *p* < 0.02; Table 1). Subsequently, a stratified analysis was performed between age and clinical stage. All patients with lung cancer were classified into young subgroup (≤ 65 years) and older subgroup (> 65 years). As shown in Fig. S2A, the cutoff value of this model could stratify these young patients into high and low-risk subgroup with significant clinical outcome (HR = 2.427 (1.465–4.019), *p* = 3.77e−04) in the training set. For the older subgroup, this model exposed the similarly predictive power in prognosis in TCGA cohort (*p* = 0.048; Fig. S2A). Next, this new model was further assessed in patients with different clinical stage (Stage I and ≥ Stage II) in TCGA data. Those patients from each group were subclassified into high and low-risk subgroup with significantly different prognosis (*p* < 0.03; Fig. S2A). The prognosis of patients with high-risk scores was notably worse than those with low-risk scores in both Stage I group (HR = 1.896 (0.981–3.664), *p* = 0.029) and ≥ Stage II group (HR = 1.241 (0.931–1.655), *p* = 0.024; Fig. S2A). For another three testing sets, the prognosis was analogous with the result above in patients within different age subgroups and clinical stage subgroups (Fig. S2B–D) except for Age ≤ 65 and Stage I in GSE72094 (*p* > 0.05; Fig. S2D), where the statistical results could not reach significance. All these results showed that the prognostic power of this 2-gene model was independent of other clinicopathological factors for patients with lung cancer.

**Table 1:** Univariate and multivariate analyses of two inflammation-related gene models in patients with LUAD

Variables	Univariate analysis			Multivariate analysis		
	HR	95% CI	*p*-value	HR	95% CI	*p*-value
TCGA						
Model	0.61	0.43–0.87	0.006	0.61	0.43–0.87	0.006
Age	0.70	0.49–0.99	0.047	0.70	0.49–0.99	0.047
Stage	0.36	0.25–0.52	< 0.001	0.36	0.25–0.52	< 0.001
Smoked	0.86	0.59–1.26	0.439			
GSE31210						
Model	0.30	0.12–0.77	0.012	0.30	0.12–0.77	0.012
Age	0.40	0.20–0.80	0.010	0.40	0.20–0.80	0.010
Stage	0.24	0.12–0.48	< 0.001	0.24	0.12–0.48	< 0.001
Smoked	1.72	0.87–3.41	0.119			
GSE68465						
Model	0.69	0.53–0.90	0.006	0.69	0.53–0.90	0.006
Age	0.65	0.50–0.84	< 0.001	0.65	0.50–0.84	< 0.001
Stage	0.43	0.30–0.60	< 0.001	0.43	0.30–0.60	< 0.001
Smoked	1.27	0.79–2.02	0.323			
GSE72094						
Model	0.59	0.39–0.88	0.010	0.59	0.39–0.88	0.010
Age	0.70	0.46–1.08	0.105			
Stage	0.38	0.26–0.56	< 0.001	0.38	0.26–0.56	< 0.001
Smoked	1.37	0.60–3.14	0.459			

## Discussion

In the past few years, there has been a gradual increase in mRNA studies associated with the progression of LUAD. Existing research indicates that inflammatory genes play a crucial role in regulating immune responses and are significant in the immunotherapy of LUAD. However, there is a relative scarcity of risk diagnostic studies on inflammatory-related genes in large datasets, often characterized by a plethora of redundant genes. Therefore, a robust and reliable inflammatory gene risk analysis model is needed for both research and clinical samples to accurately identify and stratify LUAD subtypes.

To explore the biological mechanisms of inflammation-related genes, we identified 2 subgroups independent of clinical factors by unsupervised clustering and featured inflammatory cancer genes. Through enrichment analysis, we discovered that the signaling pathways in patients with favorable prognosis were associated with cancer immune response, while the group with poorer survival was correlated with oncogenic signaling, such as MAPK signaling pathway, PI3K-AKT-MTOR signaling, VEGF signaling pathway, ERBB signaling pathway, and so on. Consistent results were obtained in GSVA and immune infiltration analyses, providing a comprehensive explanation for the relationship between different subtypes and prognosis.

To solve the problem of redundant genes, this study was based on the principle of "less likely genes". We used algorithms, such as Lasso survival analysis, enrichment analysis, and GSVA, to select the optimal inflammatory survival gene set (*MS4A1* [48–51], *MS4A2* [40, 52, 53]) between the two subgroups. Then, we developed a two inflammation-related mRNAs model as a LUAD risk prediction model based on a multifactorial Cox regression method, which was able to provide an effective prediction of the risk status of LUAD.

To further demonstrate the generalizability of this 2-gene model, we validated the model in another three independent validation sets. All datasets were categorized into two groups based on the median risk score in the training set, with the high-risk group with a poorer prognosis. We then evaluated the association between this new 2-gene model and other important clinical factors, demonstrating the independence of this model in prognosis prediction through the univariate and multivariate Cox regression. The results indicated that our proposed prognostic model has excellent risk prediction ability and practical value. All these findings confirmed the model's effectiveness in stratifying accurately the risk status of LUAD patients. In addition, it is noteworthy that the model is more streamlined of gene number and focuses on specific family genes and biological functions compared to recently published prognostic models for LUAD patients [42–47], which enhanced clinical applicability may provide a novel view for clinical prognostic diagnosis (*p* < 0.01).

Importantly, it is unknown whether this 2-gene model could predict the prognosis with the similar power in lung cancer patients from different hospitals in China because this predictive model was developed in the TCGA dataset. Another limitation of this study is that this new model should be further validated in the prospect cohorts. In addition, this study conducted functional enrichment analysis on HALLMARK and KEGG pathways to infer the biological functions of different subgroups of LUAD patients. However, these findings should be validated through biological experiments.

Generally, our study identified two important prognostic mRNAs (*MS4A1*, *MS4A2*) that were significantly altered between high- and low-risk patients with lung cancer and developed a risk model independent of the clinical factors. The model succeeded in robustly predicting patient risk subtypes in multiple testing sets. Especially, this is a risk prediction model developed for the assessment of LUAD patients by inflammation-related genes signature.

## Supplementary Information

Below is the link to the electronic supplementary material.

Supplementary file1 Filtering the optimum mRNAs to develop the 2-gene model. A) PCA plot showed two clusters from 310 LUAD patients. B) Scatter plot showed the 301 cancer genes of log2 expression from 310 DEGs. C) Determination of optimal lambda values for differentially expressed genes (DEGs) associated with survival (PNG 1192 KB)

Supplementary file2 Independence analysis of the 2-gene model between high and low-risk subtype stratified by age and clinical stage through Kaplan–Meier survival analyses. A) All patients with LUAD in the training set. B-D) All patients with LUAD in another 3 independent validation sets (PNG 463 KB)

Supplementary file3 (XLSX 10 KB)

## Data Availability

The datasets during and/or analyzed during the current study are available from the corresponding author on reasonable request.

## Abbreviations

LUAD: Lung adenocarcinoma
TCGA: The Cancer Genome Atlas
HR: Hazard ratios
CI: Confidence intervals
GSEA: Gene set enrichment analysis
GSVA: Gene set variation analysis
NSCLC: Non-small cell lung cancer
OS: Overall survival
PLR: Platelet–lymphocyte ratio
NLR: Neutrophil–lymphocyte ratio
LMR: Lymphocyte–monocyte ratio
PCA: Principal component analysis
DEGs: Differentially expressed genes
MSigDB: Molecular signature database
