# Supplementary figures and images for: Two inflammation-related genes model could predict risk in prognosis of patients with lung adenocarcinoma
Source: Clin Transl Oncol. 2025 Mar 7;27(8):3386–98. doi: 10.1007/s12094-025-03861-w (PMC12259757; doi:10.1007/s12094-025-03861-w)

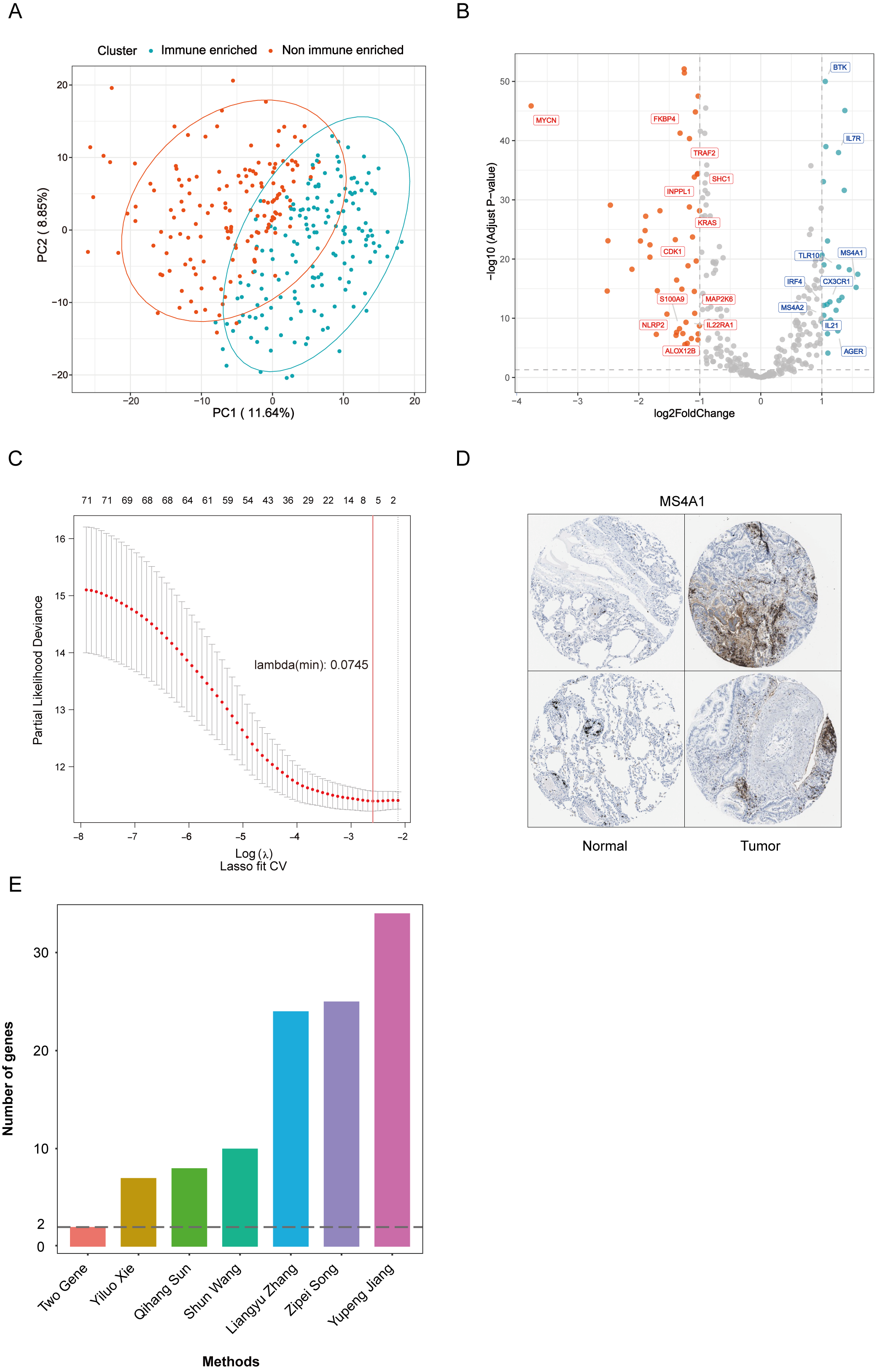

Supplement: Supplementary file 1 — Supplementary file1 Filtering the optimum mRNAs to develop the 2-gene model. A) PCA plot showed two clusters from 310 LUAD patients. B) Scatter plot showed the 301 cancer genes of log2 expression from 310 DEGs. C) Determination of optimal lambda values for differentially expressed genes (DEGs) associated with survival (PNG 1192 KB) [file 12094_2025_3861_MOESM1_ESM.png]

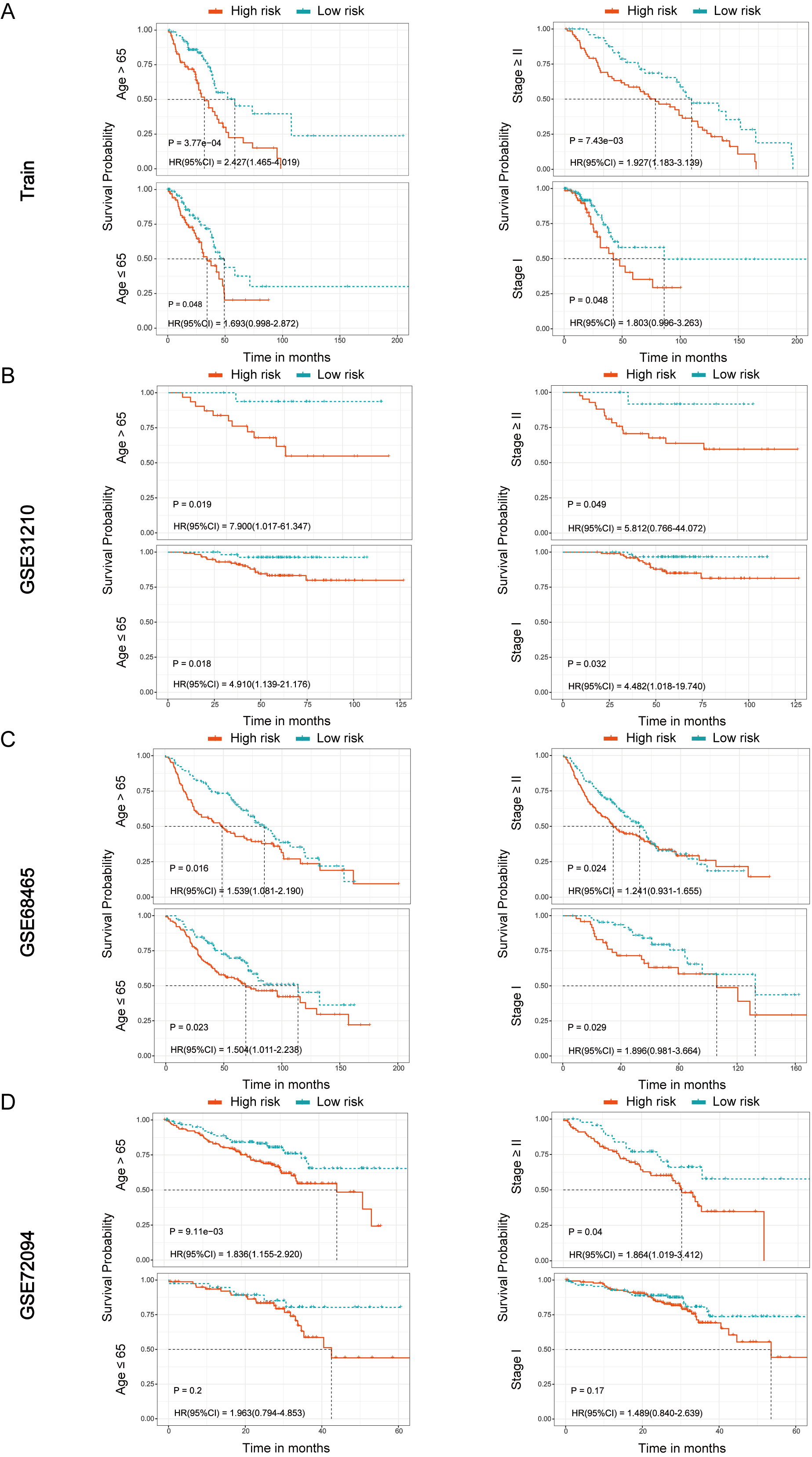

Supplement: Supplementary file 2 — Supplementary file2 Independence analysis of the 2-gene model between high and low-risk subtype stratified by age and clinical stage through Kaplan–Meier survival analyses. A) All patients with LUAD in the training set. B-D) All patients with LUAD in another 3 independent validation sets (PNG 463 KB) [file 12094_2025_3861_MOESM2_ESM.png]
